# Patterns of cortical thinning in nondemented Parkinson's disease patients

**DOI:** 10.1002/mds.26590

**Published:** 2016-04-20

**Authors:** Carme Uribe, Barbara Segura, Hugo Cesar Baggio, Alexandra Abos, Maria Jose Marti, Francesc Valldeoriola, Yaroslau Compta, Nuria Bargallo, Carme Junque

**Affiliations:** ^1^ Department of Psychiatry and Clinical PsychobiologyUniversity of BarcelonaBarcelonaCataloniaSpain; ^2^ Centro de Investigación Biomédica en Red sobre Enfermedades Neurodegenerativas (CIBERNED), Hospital Clínic de BarcelonaBarcelonaCataloniaSpain; ^3^ Movement Disorders Unit, Neurology Service, Hospital Clínic de BarcelonaBarcelonaCataloniaSpain; ^4^ Centre de Diagnostic per la Imatge, Hospital ClinicBarcelonaCataloniaSpain; ^5^ Institute of Biomedical Research August Pi i Sunyer (IDIBAPS)BarcelonaCataloniaSpain

**Keywords:** Parkinson disease, cluster analysis, neuropsychology, magnetic resonance imaging, cortical atrophy

## Abstract

**Background:**

Clinical variability in the Parkinson's disease phenotype suggests the existence of disease subtypes. We investigated whether distinct anatomical patterns of atrophy can be identified in Parkinson's disease using a hypothesis‐free, data‐driven approach based on cortical thickness data.

**Methods:**

T1‐weighted 3‐tesla MRI and a comprehensive neuropsychological assessment were performed in a sample of 88 nondemented Parkinson's disease patients and 31 healthy controls. We performed a hierarchical cluster analysis of imaging data using Ward's linkage method. A general linear model with cortical thickness data was used to compare clustering groups.

**Results:**

We observed 3 patterns of cortical thinning in patients when compared with healthy controls. Pattern 1 (n = 30, 34.09%) consisted of cortical atrophy in bilateral precentral gyrus, inferior and superior parietal lobules, cuneus, posterior cingulate, and parahippocampal gyrus. These patients showed worse cognitive performance when compared with controls and the other 2 patterns. Pattern 2 (n = 29, 32.95%) consisted of cortical atrophy involving occipital and frontal as well as superior parietal areas and included patients with younger age at onset. Finally, in pattern 3 (n = 29, 32.95%), there was no detectable cortical thinning. Patients in the 3 patterns did not differ in disease duration, motor severity, dopaminergic medication doses, or presence of mild cognitive impairment.

**Conclusions:**

Three cortical atrophy subtypes were identified in nondemented Parkinson's disease patients: (1) parieto‐temporal pattern of atrophy with worse cognitive performance, (2) occipital and frontal cortical atrophy and younger disease onset, and (3) patients without detectable cortical atrophy. These findings may help identify prognosis markers in Parkinson's disease. © 2016 The Authors. Movement Disorders published by Wiley Periodicals, Inc. on behalf of International Parkinson and Movement Disorder Society

Parkinson's disease (PD) is associated with progressive cognitive impairment and cortical atrophy.^1^ Clinical variability in PD suggests the existence of disease subtypes. A review of cluster analysis studies concluded that there is clear evidence of 2 clinical profiles: one with old‐age onset and rapid disease progression and another of younger age at onset and slower progression.^2^ Recently, Fereshtehnejad and colleagues^3^ identified the following 3 subtypes while considering clinical and cognitive variables: motor/slow progression, diffuse/malignant, and intermediate. Patients with diffuse/malignant PD more often had mild cognitive impairment (MCI) and showed faster cognitive deterioration.

Considering the relevance of cognitive status in the risk of dementia, cluster analysis has also been used to describe subtypes according to neuropsychological performance. Dujardin and colleagues^4^ described 2 groups. One group was composed of cognitively intact subjects and patients with lower scores on working memory, verbal episodic memory, and executive functions, although within the normal range. The second group included PD patients with varying degrees of impairment in all cognitive domains. The identification of PD subtypes based on objective and replicable measures is critical to define targets for possible future treatments that improve the prognosis of PD. To our knowledge, no previous studies used hypothesis‐free, data‐driven cluster analysis of objective measures such as structural magnetic resonance imaging (MRI) data to identify subtypes of cortical atrophy in PD patients.

The main objective of this study was to examine cortical thickness in a large sample of nondemented PD patients using cluster analysis to determine whether distinct anatomical patterns can be established and whether different patterns are associated with distinct cognitive profiles.

## Methods

### Participants

The study sample included 121 PD patients recruited from the Parkinson's Disease and Movement Disorders Unit, Hospital Clínic (Barcelona, Spain), and 49 healthy controls (HC) from the Aging Institute in Barcelona. All participants underwent comprehensive neuropsychological and MRI evaluations. Inclusion criteria for patients were (i) fulfilling UK PD Society Brain Bank diagnostic criteria for PD^5^ and (ii) no surgical treatment with deep‐brain stimulation. Exclusion criteria for all participants were (i) dementia according to Movement Disorders Society criteria,^6^ (ii) Hoehn and Yahr (H&Y) scale^7^ score > 3, (iii) young‐onset PD, (iv) age < 50 years, (v) severe psychiatric or neurological comorbidity, (vi) low global intelligence quotient estimated by the Vocabulary subtest of the Wechsler Adult Intelligence Scale 3rd edition (scalar score ≤ 7), (vii) Mini Mental State Examination (MMSE)^8^ score below 25, (viii) claustrophobia, (ix) pathological MRI findings other than mild white‐matter hyperintensities in the FLAIR sequence, and (x) MRI artifacts.

A total of 88 PD patients and 31 HC were selected. The following participants were excluded from the study: 12 patients and 8 HC because of dementia or another neurological disease, 6 patients for psychiatric comorbidity, 1 patient with an H&Y score of > 3, 1 patient with young‐onset PD, 3 patients and 1 HC with low IQ scores, 2 patients for claustrophobia, 3 HC who did not complete the neuropsychological assessment, and 2 patients and 2 HC with MRI artifacts. We also excluded 4 patients and 3 HC aged younger than 50 years, and 2 patients and 1 HC because they were outliers in cluster analyses, constituting a cluster by themselves.

Motor symptoms were assessed with the Unified Parkinson's Disease Rating Scale, motor section (UPDRS‐III).^9^ All PD patients were taking antiparkinsonian drugs that consisted of different combinations of l‐dopa, cathecol‐O‐methyltransferase inhibitors, monoamine oxidase inhibitors, dopamine agonists, and amantadine. To standardize the doses, the l‐dopa equivalent daily dose (LEDD)^10^ was calculated.

Written informed consent was obtained from all study participants after a full explanation of the procedures. The study was approved by the institutional Ethics Committee for Clinical Research.

### Neuropsychological Tests

We used a neuropsychological battery following the Movement Disorders Society task force recommendations^11^; bar language, for which a single measure was used; and executive functions, for which phonemic and semantic verbal fluency were used as 2 distinct proxies. Supplementary Methods 1 describes the tests used in the neuropsychological assessment.

Facial emotion recognition was assessed with the Ekman 60 Faces Test.^12^ Emotion recognition has been described to be impaired in PD patients, and the Ekman test has shown sensitivity to the integrity of the orbitofrontal cortex (OFC) in PD.^13^ Neuropsychiatric symptoms were evaluated with the Beck Depression Inventory‐II,^14^ Starkstein's Apathy Scale,^15^ and Cumming's Neuropsychiatric Inventory.^16^

### Image Analysis

MRI data were acquired with a 3T scanner (MAGNETOM Trio, Siemens, Germany). The scanning protocol included high‐resolution 3‐dimensional T1‐weighted images acquired in the sagittal plane (TR = 2300 ms, TE = 2.98 ms, TI = 900 ms, 240 slices, FOV = 256 mm; 1 mm isotropic voxel) and an axial FLAIR sequence (TR = 9000 ms, TE = 96 ms).

Cortical thickness was estimated using the automated FreeSurfer stream (version 5.1, http://surfer.nmr.harvard.edu). Detailed descriptions of FreeSurfer procedures are in Supplementary Methods 2.

### Cluster Analysis

**Figure 1:**
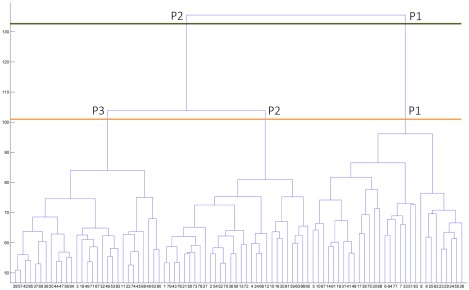
Dendrogram of PD patients clustered according to vertex‐by‐vertex information of cortical thickness. **T**he distance along the *y* axis represents the similarity between clusters so that the shorter the distance, the greater the similarity. Numbers on the horizontal axis represent the 88 PD patients included in the cluster analysis. P1, Pattern 1; P2, Pattern 2; P3, Pattern 3. [Color figure can be viewed in the online issue, which is available at wileyonlinelibrary.com.]

MATLAB (release 2014b, The MathWorks, Inc., Natick, Massachusetts) was used to perform an agglomerative hierarchical cluster analysis using whole‐brain cortical thickness vertex information for each of the 88 PD patients. Each patients' cortical surface data included 327,684 vertices. This technique produces hierarchical representations, and clusters at each hierarchical level are created by merging clusters at the next lower level. In hierarchical cluster analysis, there is no need to specify the number of clusters a priori because grouping is based on the dissimilarity between groups of observations. To control for variations in global atrophy between patients, vertices were normalized using whole‐brain mean cortical thickness.^17^, ^18^ Ward's clustering linkage method^17^, ^18^, ^19^ was used to combine pairs of clusters at each step while minimizing the sum of square errors from the cluster mean. Each of the 88 patients was placed in their own cluster and then progressively clustered with others. Cluster analysis results are shown as a dendrogram (Fig. 1).

### Statistical Analysis

Intergroup cortical thickness comparisons were performed using a vertex‐by‐vertex general linear model with FreeSurfer. The model included cortical thickness as a dependent factor and group as an independent factor. Age and education were considered as nuisance covariates when they were significantly different between the groups being compared (Table 1). All results were corrected for multiple comparisons using precached clusterwise Monte Carlo simulation with 10,000 iterations. Reported cortical regions reached a 2‐tailed corrected significance level of *P* < .05.

**Table 1:** Demographic and clinical characteristics at the 3‐cluster level

	PD subtypes			HC (n = 31)	
	Pattern 1 (n = 30)	Pattern 2 (n = 29)	Pattern 3 (n = 29)		Test stats, *P* value
Sex, male, n (%)	15 (50.0)	20 (69.0)	16 (55.2)	16 (51.6)	2.667, .446^a^
Age, y, mean (SD)	70.60 (9.6)	58.03 (8.9)	63.48 (9.5)	64.32 (8.5)	9.401, < .0001^b^, ^f^, ^g^
Education, y, mean (SD)	7.77 (4.8)	13.55 (5.5)	10.55 (4.0)	11.03 (4.2)	7.622, < .0001^b^, ^d^, ^f^
MMSE, mean (SD)	28.57 (1.4)	29.24 (0.9)	29.31 (0.9)	29.68 (0.5)	6.944, < .0001^c^, ^d^
Disease duration, y, mean (SD)	8.77 (6.6)	8.36 (5.7)	6.83 (4.6)	NA	0.949, .391^b^
Age of onset, y, mean (SD)	61.83 (12.7)	49.67 (8.3)	56.66 (10.3)	NA	9.710, < .0001^c^, ^f^, ^h^
Early PD, 5 y n, (%)	12 (40.0)	11 (37.9)	14 (48.3)	NA	0.715, .699^a^
BDI, mean (SD)	13.67 (5.7)	8.88 (6.8)	9.61 (5.7)	6.03 (5.7)	7.888, < .0001^b^, ^d^, ^f^
Apathy, mean (SD)	15.11 (7.9)	11.60 (7.1)	11.29 (6.0)	8.38 (5.1)	4.958, .003^c^, ^d^
NPI, mean (SD)	6.59 (7.8)	4.41 (8.2)	6.21 (6.5)	1.52 (3.2)	3.242, .025^c^, ^d^, ^e^
Visual hallucinations, n (%)	6 (20.0)	6 (22.2)	5 (17.2)	0 (0)	7.900, .245^a^
UPDRS part III, mean (SD)	18.07 (9.1)	15.17 (11.6)	13.07 (8.4)	NA	1.945, .149^b^
Hoehn & Yahr stage, n 1/1.5/2/2.5/3	2/3/16/4/5	9/2/13/3/2	11/0/14/1/3	NA	12.262, .140^a^
LEDD, mg, mean (SD)	764.63 (388.3)	930.52 (576.4)	718.00 (493.9)	NA	1.503, .228^b^
Total MCI, n (%)	20 (66.7)	14 (48.3)	11 (37.9)	NA	5.015, .081^a^
Visuospatial functions, n (%)	10 (33.3)	9 (31.0)	7 (24.1)	NA	0.645, .724^a^
Executive functions, n (%)	16 (53.3)	6 (20.7)	6 (20.7)	NA	9.712, .008^a^
Memory, n (%)	14 (46.7)	11 (37.9)	9 (31.0)	NA	1.529, .466^a^
Attention and WM, n (%)	20 (66.7)	17 (58.6)	14 (48.3)	NA	2.055, .358^a^
Language, n (%)	2 (6.7)	3 (10.3)	2 (6.9)	NA	0.339, .844^a^

Apathy, Starkstein's Apathy Scale; BDI, Beck Depression Inventory‐II; HC, healthy controls; LEDD, l‐dopa equivalent daily dose; MCI, Mild Cognitive Impairment; MMSE, Mini‐Mental State Examination; NA, not applicable; NPI, Cumming's Neuropsychiatric Inventory; PD, Parkinson's disease; UPDRS III, Unified Parkinson's Disease Rating Scale motor section; WM, working memory.

Data are presented as mean (standard deviation) (continuous) or frequencies (categorical).

a The Chi‐squared test was used.

b Analysis of variance followed by Bonferroni post hoc test was used.

c Analysis of variance followed by Tamhane (T2) post hoc test was used.

d Significant post hoc differences (*P* < .05) between HC and pattern 1.

e Significant post hoc differences (*P* < .05) between HC and pattern 3.

f Significant post hoc differences (*P* < .05) between pattern 1 and pattern 2.

g Significant post hoc differences (*P* < .05) between pattern 1 and pattern 3.

h Significant post hoc differences (*P* < .05) between pattern 2 and pattern 3.

Demographic, neuropsychological, and clinical statistical analyses were conducted using IBM SPSS Statistics 20.0 (IBM Corp., Armonk, New York). We tested for group differences in demographic and clinical variables as well as in neuropsychological performance between HC and PD patient subtypes using an analysis of variance with a Bonferroni or Tamhane post hoc test when analyzing quantitative variables and the Pearson chi‐square test when analyzing categorical variables. For comparisons between the collapsed PD group and HC we used the Student *t* test. Neuropsychological test scores were calculated as *z* scores and adjusted for age, years of education, and sex as previously described.^20^

MATLAB was used to perform principal component analysis (PCA) to validate the classification obtained from the cluster analysis. PCA is a multivariate method that can detect correlations in a set of variables.^21^ After discarding vertices with values of zero and vertices that correlated highly with others, PCA was performed with 4,150 vertices.^22^

## Results

### Demographic and Clinical Characteristics

Compared with HC, the collapsed PD sample had significantly lower MMSE scores as well as more severe depression, apathy, and global neuropsychiatric symptoms (all *P* ≤ .001) (Supplementary Table 1).

### PD Subtypes According to Cluster Analysis

Models with 2 and 3 clusters were selected as possible solutions. Detailed information about the 2‐cluster and 4‐cluster solutions is included as supplementary results (see Supplementary Result 1 and Supplementary Tables 2, 3, and 4).

**Figure 2:**
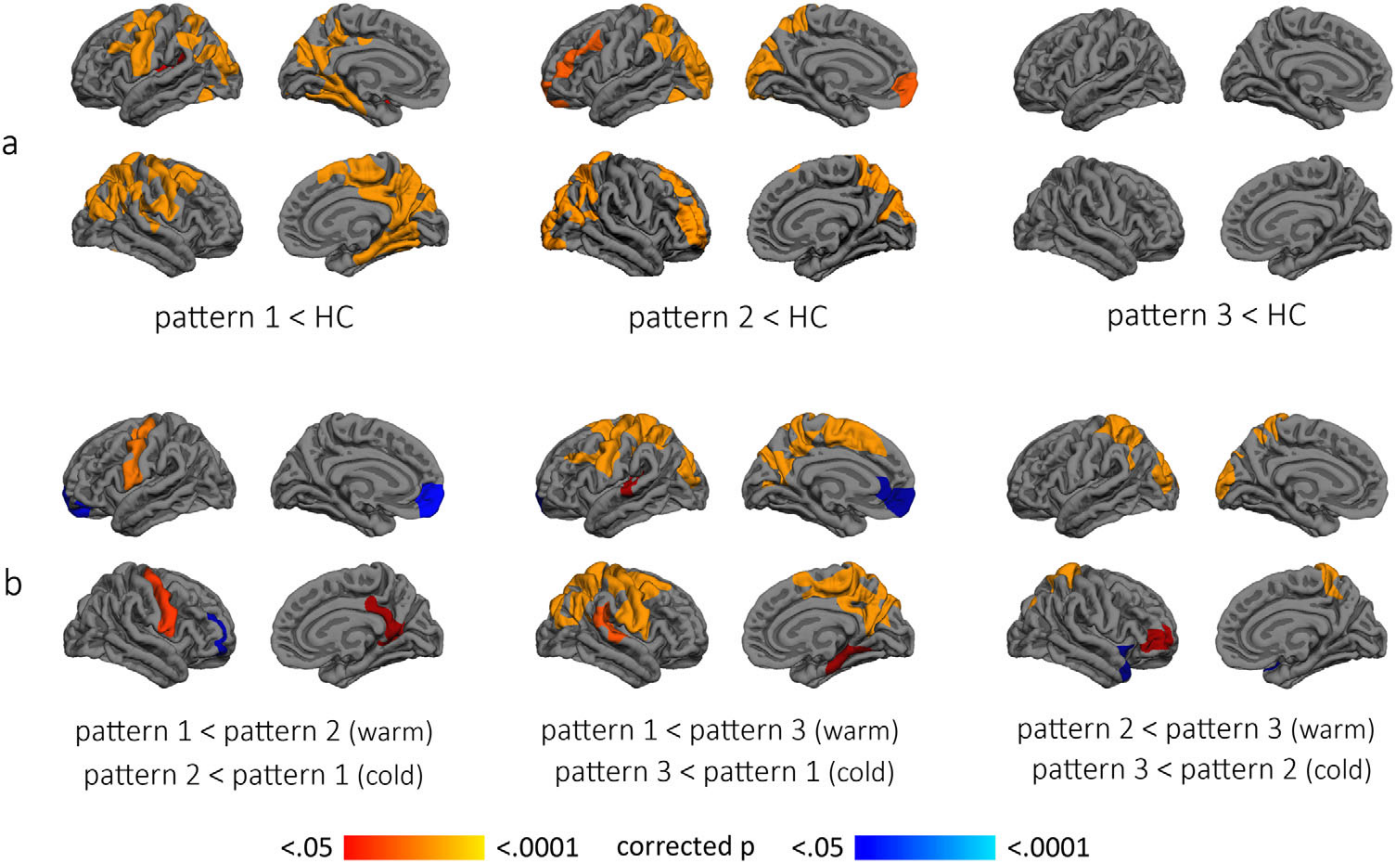
Cortical atrophy patterns at 3‐cluster level. **a**: Color maps indicate significant thinning when compared with healthy controls. **b**: Color maps indicate significant differences in thickness between the 3 patterns. Results were corrected by Monte Carlo simulation. HC, healthy controls. [Color figure can be viewed in the online issue, which is available at wileyonlinelibrary.com.]

At the 3‐cluster level (Fig. 2a), 3 patterns of cortical thickness were identified. PD patients included in pattern 1 (n = 30, 34.09%) showed reduced cortical thickness when compared with HC in lateral and medial regions bilaterally, including the precentral gyrus, inferior and superior parietal areas, cuneus, posterior cingulate gyrus, and parahippocampal gyrus. Years of education were controlled for when comparing pattern 1 with HC (see Table 1). Pattern 2 included patients (n = 29, 32.95%) with cortical atrophy in bilateral superior parietal and occipital areas and bilateral frontal regions such as the middle frontal, orbitofrontal, and right anterior superior frontal. Patients in the third cluster, pattern 3 (n = 29, 32.95%), showed no significant cortical thinning when compared with HC.

Comparisons between patients in different patterns also showed significant differences (see Fig. 2b). PD patients included in pattern 1 showed cortical thinning in the posterior cingulate/isthmus of the cingulate gyrus and precuneus as well as precentral gyrus in comparison with pattern 2 patients. Pattern 2 patients showed cortical thinning in dorsolateral and orbital frontal regions when compared with pattern 1 patients. Age and years of education were controlled for when comparing these two groups (Table 1).

Pattern 1 patients showed significant cortical thinning in lateral and medial regions bilaterally, including the precentral gyrus, inferior and superior parietal areas, cuneus, posterior cingulate gyrus, and parahippocampal gyrus when compared with pattern 3 patients. On the other hand, when compared with pattern 1 patients, pattern 3 patients showed cortical thinning in the left medial OFC. Age was controlled for when comparing these groups (Table 1).

Finally, pattern 2 patients showed cortical thinning in the superior parietal and occipital areas and in the left dorsolateral frontal cortex in comparison with pattern 3 patients.

### Demographic and Clinical Characteristics

There were no significant differences in motor disease severity as measured by the UPDRS‐III, H&Y, and LEDD or disease duration between groups at the 3‐cluster level. Patients in pattern 1 had lower MMSE scores than HC and were less educated than both HC and pattern 2 patients. Patients in pattern 2 were younger at PD onset than patients in patterns 1 and 3. Regarding psychiatric symptoms, patients in pattern 1 were more depressed than both HC and pattern 2 patients and more apathetic than HC. Patients in patterns 1 and 3 had more severe global neuropsychiatric symptoms than HC (see Table 1).

### Cognitive Profiles of PD Subtypes

**Figure 3:**
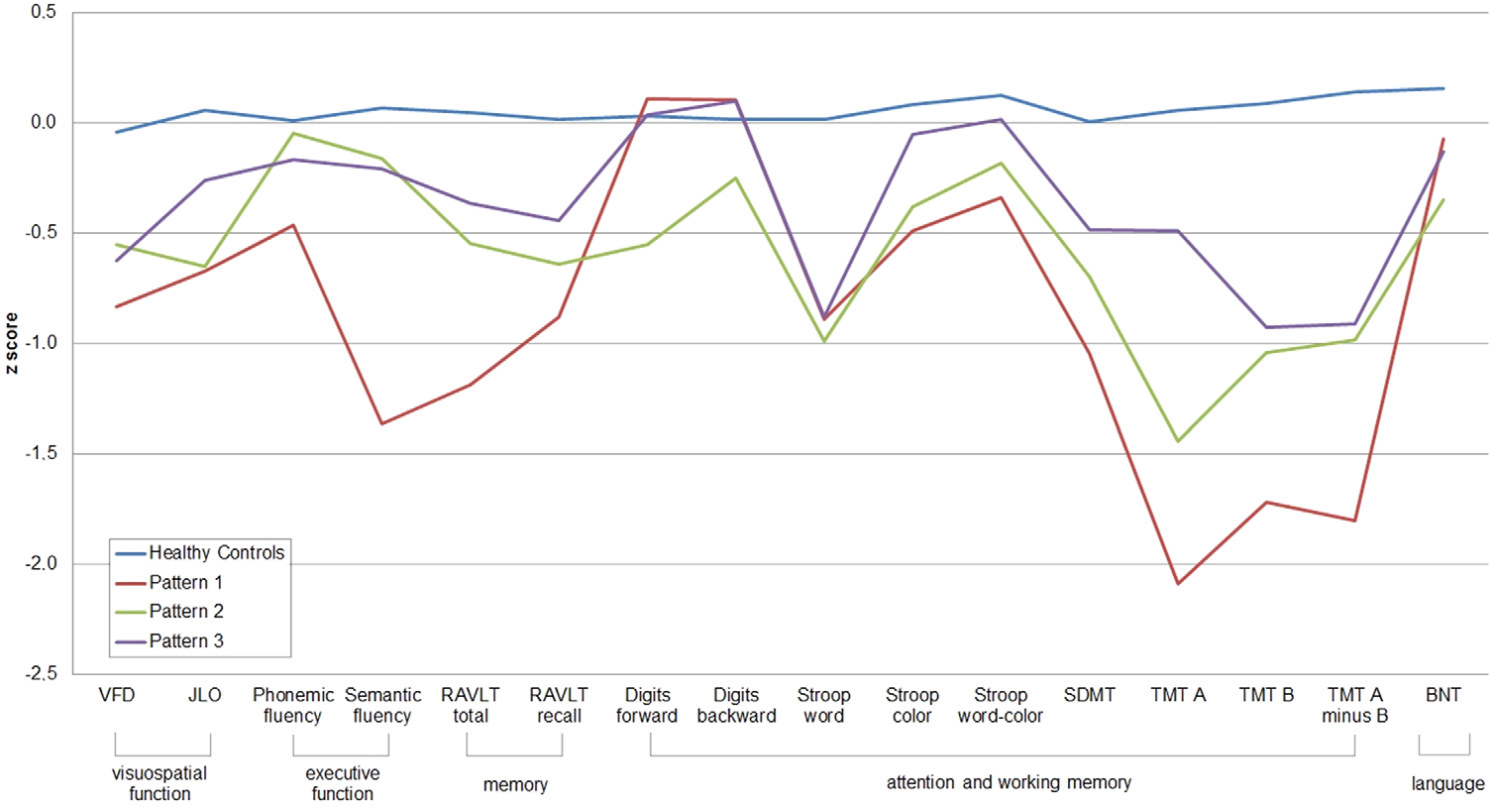
Neuropsychological profile at the 3‐cluster level. Neuropsychological profiles for healthy controls in green, pattern 1 in blue, pattern 2 in red, and pattern 3 in purple. Data are presented as *z* scores. Lower *z* scores indicate worse performance. BNT, Boston Naming Test; JLO, Judgment of Line Orientation Test; RAVLT total, Rey Auditory Verbal Learning Test total; RAVLT recall, Rey Auditory Verbal Learning Test recall after 30 minutes; SDMT, Symbol Digits Modalities Test; TMTA, Trail Making Test Part A; TMTB, Trail Making Test Part B; TMTA minus B, Trail Making Test A minus B; VFD, Visual Form Discrimination Test. [Color figure can be viewed in the online issue, which is available at wileyonlinelibrary.com.]

Figure 3 summarizes the cognitive profiles of patients in the 3 patterns. When compared with HC, patients in pattern 1 displayed significantly worse performance in Visual Form Discrimination Test, Judgment of Line Orientation Test (JLO), semantic fluency, Rey Auditory Verbal Learning Test total learning and delayed recall, Stroop (Word and Color), Symbol Digits Modalities Test (SDMT), Trail Making Test Part A (TMTA); Trail Making Test Part B (TMTB), and Trail Making Test A minus B (TMTA minus B). Performance in the semantic fluency test was significantly worse in pattern 1 patients than in the 3 other groups (HC and patients in patterns 2 and 3). Pattern 2 patients differed from HC in the JLO, Stroop Word test, SDMT, and TMTB and TMTA minus TMTB tests. Patients in pattern 3 scored significantly lower than HC in the Stroop Word test. The means (SD) of the *z* scores are shown in Supplementary Table 5. There were no significant differences in the proportion of patients with MCI between groups (Table 1).

### Emotion Recognition

There were no significant intergroup differences in overall facial emotion recognition. Analyzing individual emotion recognition, post hoc testing showed that the accuracy in identifying sadness in pattern 2 patients was significantly lower than in the HC group (Bonferroni corrected *P* = .044) (Table 2).

**Table 2:** Results from emotion recognition tests at the 3‐cluster level

	PD subtypes			HC, n = 31, mean (SD)	
	Pattern 1, n = 30, mean (SD)	Pattern 2, n = 29, mean (SD)	Pattern 3, n = 29, mean (SD)		Test stats,^a^ *P* value
Anger	−0.23 (1.0)	−0.23 (1.1)	0.00 (0.7)	0.07 (1.0)	0.762, .518
Disgust	−0.45 (1.6)	−0.43 (1.1)	−0.50 (1.0)	0.09 (0.9)	1.513, .216
Fear	0.00 (0.8)	0.07 (0.8)	−0.04 (1.0)	−0.07 (1.0)	0.124, .946
Sadness	−0.19 (1.1)	−0.53 (0.9)	−0.27 (0.7)	0.14 (0.7)	2.587, .057^b^
Happiness	−0.18 (1.6)	−0.58 (1.9)	−0.22 (1.1)	−0.11 (1.0)	0.526, .665
Surprise	−0.40 (1.4)	−0.11 (1.1)	0.06 (0.9)	0.04 (0.9)	1.040, .378
Total score	−0.12 (0.7)	−0.03 (0.6)	0.02 (0.5)	0.02 (1.1)	0.209, .890

HC, healthy controls; PD, Parkinson's disease; SD, standard deviation.

Results of the Ekman 60 Faces Test, presented in *z* scores.

a Analysis of variance.

b Significant differences between HC and pattern 2 in Bonferroni post hoc test (*P* < .05).

### PCA Validation

The patterns identified through PCA were similar to those obtained with cluster analysis. Details and representation of the PCA results are shown in Supplementary Results 2 and Supplementary Figure 1.

## Discussion

The main finding of this study is that data‐driven analysis can classify PD according to patterns of cortical degeneration. We identified a 3‐cluster solution including (1) mainly parietal‐temporal atrophy, (2) frontal and occipital atrophy, and (3) nonatrophic PD subtypes. To our knowledge, this is the first study to obtain cortical thinning patterns through cluster analysis in nondemented PD, showing different PD subtypes.

Previous neuroimaging studies assessed cortical atrophy at different clinical stages of PD and showed inconsistent results. Cortical thinning has been identified in de novo,^23^ nondemented,^24^ MCI,^25^, ^26^, ^27^, ^28^ and demented PD patients.^29^ However, the heterogeneity of these results prevents the identification of specific cortical patterns of degeneration in PD progression. The existence of different cortical atrophy subtypes in nondemented PD patients, identified using a hypothesis‐free approach, should help clarify the inconsistency of previous results and help study different patterns of structural degeneration over time.

Patients grouped in pattern 1 showed cortical atrophy in dorsal and medial cortices bilaterally, mainly involving parieto‐temporal regions. This pattern partially overlapped with the cortical atrophy previously described in nondemented PD patients^24^ and patients with MCI.^28^ In this previous study, however, PD patients with MCI also showed cortical atrophy in prefrontal and lateral temporal regions.^28^ Different methodological approaches might explain the discrepant results. The patterns identified in the present study were based on objective anatomical data without prior patient classification according to the presence or absence of MCI.

Interestingly, we identified a second cortical thinning pattern, specifically involving frontal (medial OFC and rostral middle frontal) and occipital (cuneus and lateral occipital) atrophy. Similar to pattern 1, patients in this group displayed inferior and superior parietal atrophy, but medial parietal and temporal regions were preserved. A similar pattern of degeneration has been identified in studies of brain metabolism in PD patients. Occipital and frontal (18)F‐fluorodeoxyglucose positron emission tomography (PET) hypometabolism has been reported as a signature of cognitive impairment in PD.^30^, ^31^, ^32^ Cortical hypoperfusion, mainly in frontal, parietal, and occipital regions, has also been identified using arterial spin labeling perfusion MRI in nondemented PD.^33^ Furthermore, metabolic single‐photon emission computed tomography and PET studies have suggested the existence of widespread brain metabolic changes associated with cognitive impairment involving multiple domains^34^, ^35^, ^36^ and with single‐domain nonamnestic deficits.^36^

To date, atrophy in occipital and frontal regions has not been evidenced using other structural MRI techniques such as voxel‐based morphometry.^31^, ^33^ In line with our results, previous studies seem to indicate that cortical thickness measures are more sensitive to occipital cortical atrophy in PD.^37^, ^38^

The pathological meaning of the differences between patterns identified in our study is unclear. Prior pathological findings in PD, including Lewy neurites and Lewy bodies containing ubiquitin and α‐synuclein aggregations, provide a general progression of brain alterations from the medulla and olfactory bulb to the midbrain, diencephalic nuclei, and finally to the neocortex following Braak staging.^39^ Braak's classification has been seen to correlate with neurological deficits in patients with early‐onset PD and long disease duration.^40^ Conversely, it has also been stated that Braak staging is not related to clinical severity and cognitive impairment.^41^ Thus, the relationship between the presence of α‐synuclein aggregates and cognitive deficits in PD remains controversial. Recent studies have shown an increase in the severity of α‐synuclein pathology in the basal forebrain and hippocampus in combination with more widespread degeneration of cortical dopaminergic and cholinergic pathways in demented PD patients.^42^ On the other hand, Alzheimer's disease–type pathology has been highlighted as an important cofactor in the progression of cognitive impairment in PD^43^, ^44^ as well as other pathological findings such as cerebrovascular disease and hippocampal sclerosis (see Halliday and colleagues^45^ for a review). In our opinion, our results might be related to abnormal protein deposition, including α‐synucleinopathy and Alzheimer's disease–type pathology, as has been shown in previous neuropathological and Pittsburgh Compound‐B (PiB) PET studies.^46^ A neuropathological study of a large sample of demented PD patients showed that all patients had abnormal cortical synuclein aggregates, and 60% also had abnormal amyloid‐β deposits.^46^ In one PET study of cognitively impaired PD patients, abnormal PiB binding was observed in 17% of the patients.^47^ We could speculate that pattern 1 in our sample could be reflecting patients with abnormal amyloid‐β associated with abnormal cortical α‐synuclein deposition because patients in this group showed atrophy in the medial temporal and parietal cortices, regions reported as sensitive to progressive cortical thinning in cognitively preserved PiB + patients.^48^ Patterns 1 and 2 in our study differed in the degree of atrophy in the posterior cingulate, isthmus of the cingulate, and precuneus. In this line, it has been reported that in nondemented PD, higher PiB retention in the precuneus seems to contribute to cognitive decline over time.^49^

In addition, we identified a PD subtype without manifest cortical atrophy. This group showed no significant differences in disease duration, motor symptoms, or LEDD when compared with other PD subtypes. As such, patients in this group were not in an earlier disease stage. Interestingly, other studies reported cortical differences in gray matter atrophy between motor subtypes showing a reduction predominantly in postural‐instability and gait‐difficulty patients in comparison with tremor‐dominant patients.^50^ Our results showed no significant differences between groups in motor symptoms measured by the UPDRS. However, the specific motor profile of our groups was not evaluated in depth. Previous studies comparing HC with early PD,^23^, ^28^, ^51^ or with PD patients with and without MCI,^26^, ^27^ have often described differences that did not survive correction for multiple comparisons. In our opinion, these findings suggest the existence of a subtype of PD with slower cortical degeneration. The absence of structural changes between cognitively unimpaired de novo PD patients and HC has been reported even using techniques sensitive to subtle longitudinal changes such as tensor‐based morphometry.^52^ Longitudinal cortical thickness studies could assess whether this cortical pattern might constitute a biomarker of better cognitive prognosis.

The 3 PD subtypes identified had specific cognitive characteristics. The parietal‐temporal and occipital and frontal subtypes (patterns 1 and 2, respectively) performed significantly worse than HC on JLO, TMTB, TMTA minus TMTB, and SDMT tests, although the occipital and frontal subtype showed less pronounced impairment. In addition, the parietal‐temporal subtype also performed worse in RAVLT, Stroop Color, and TMTA and showed more severe depression and apathy symptoms than HC. However, contrary to what might have been expected, there were no differences in the proportion of patients with MCI between PD subtypes. A previous model‐based cluster analysis study using neuropsychological data^4^ also described heterogeneous cognitive impairment in PD from cognitively intact patients to very severely impaired patients with a progressive severity gradient. The authors found a group of patients within the normal range of cognitive performance, but with lower scores on working memory, verbal episodic memory, and executive functions. In addition, they found a second group of PD patients with varying degrees of impairment in all cognitive domains. Patients in the cognitively impaired cluster were older, less educated, and more apathetic than the cognitively unimpaired patients; these characteristics partially overlap with the parietal‐temporal subtype we describe. However, the cognitively impaired group in the study by Dujardin and colleagues^4^ included a wider range of cognitive deficits, from MCI to dementia, whereas our study did not include demented patients. Contrary to our results in which there were no significant differences in motor disease severity or disease duration between cluster groups, Dujardin and colleagues^4^ found that the cognitively impaired group showed more severe motor symptoms, longer disease duration, and more axial signs in comparison with cognitively unimpaired patients.

It is noteworthy that, among all the cognitive tests used, only semantic fluency specifically differentiated the parietal‐temporal pattern from other PD subtypes. We have previously shown a positive correlation between semantic fluency and medial temporal and precuneus cortical thickness.^13^ In addition, semantic fluency has been shown in population‐based longitudinal studies to be a predictor of dementia in PD.^53^, ^54^ Barker and Williams‐Gray^55^ suggested that there is a posterior cognitive syndrome with impaired semantic fluency, nondopaminergic deficits, and worse prognosis. In a recent review, Sauerbier and colleagues^56^ defined this phenotype as “Park cognitive.” Together, these results highlight the usefulness of semantic fluency as an easily administered task that should be included in the routine neuropsychological assessment to help identify this subtype of PD patients.

Focusing on the occipital and frontal subtype, patients were younger at PD onset and showed impaired recognition of sadness in facial expressions. In line with these results, voxel‐based morphometry studies showed medial OFC atrophy in younger PD patients (<70 years) when compared with HC^57^ and related it with specific cognitive deficits.^58^ Specifically, medial OFC volume has been associated with overall^58^ as well as negative facial emotion recognition in PD.^13^

Cognitive performance in the nonatrophic subtype followed a similar pattern as that in the other groups. However, only Stroop Word scores were significantly different between the nonatrophic group and HC. Similarly, as we previously mentioned, previous cluster analyses using neuropsychological data reported the existence of a PD subtype composed of cognitively intact patients and patients with lower scores (although within the normal range) on different cognitive domains commonly impaired in PD.^4^ These results could lead us to speculate the existence of a subgroup of PD patients with limited cortical atrophy with cognitive profiles similar but possibly less severe than those of patients with faster structural degeneration. Beyond the presence of α‐synuclein pathology and Alzheimer's disease–type pathology, functional deficits related to neurotransmitter deficiencies (mostly but not only dopaminergic) as well as defects involving diverse metabolic pathways (abnormal oxidative stress, gene regulation, protein degradation, and synaptic degeneration), translate as an early involvement of the cerebral cortex in PD (see Ferrer^59^ and Ferrer and colleagues^60^ for reviews). These findings might explain cognitive dysfunctions in the absence of evident structural changes. Alternatively, structural changes might be below the detection threshold of cortical thickness methods in such cases. In this vein, future fMRI connectivity studies might help to characterize the functional changes associated with the cortical thickness patterns herein identified.

Finally, none of the PD subtypes showed significant differences on the digits subtest, Stroop Word‐Color, phonemic fluency, or the BNT. The sensitivity of these tests to detect cognitive impairment in PD should be assessed in future studies using different cohorts to validate their role in recommended neuropsychological batteries. Moreover, in light of our results, it would be interesting to include other tests that could be associated with occipital and frontal atrophy, such as emotion recognition tests, in standard protocols. The early identification of these PD subtypes through cognitive and clinical characteristics could facilitate the study of different patterns of deterioration over time. In the near future, longitudinal assessments might help clarify whether the cortical atrophy patterns reported in our results are associated with clinical PD subtypes identified recently as diffuse/malignant with rapid progression to dementia, mainly motor/slow progression and intermediate.^3^

The main strength of our study is the use of cortical thickness as a main variable because this is an objective measure based on validated methods. Clustering analysis using MRI data may allow future studies using other independent cohorts to validate these patterns.

In conclusion, the cluster analysis of cortical thickness data in nondemented PD patients identified 3 subtypes consisting of (1) parieto‐temporal pattern of atrophy with significant cognitive impairment, (2) occipital and frontal cortical atrophy with younger PD onset, and (3) patients without manifest cortical atrophy. This effort to identify different PD phenotypes based on objective data could be valuable for the establishment of prognostic markers in PD.

## Author Roles

1. Research Project: A. Conception, B. Organization, C. Execution; 2. Statistical Analysis: A. Design, B. Execution, C. Review and Critique; 3. Manuscript Preparation: A. Writing the First Draft, B. Review and Critique.

C.U.: 1B, 2B, 2C, 3A, 3B

B.S.: 1A, 1B, 2A, 2B, 2C, 3A, 3B

H.C.B.: 1A, 1B, 2A, 2B, 2C, 3A, 3B

A.A.: 2B, 2C, 3B

M.J.M.: 1C, 2C, 3B

F.V.: 1C, 2C, 3B

Y.C.: 1C, 2C, 3B

N.B.: 1C, 2C, 3B

C.J.: 1A, 1B, 2A, 2C, 3B

## Financial disclosures of all authors for the preceding 12 months

C.U. was supported by a fellowship from 2014, Spanish Ministry of Economy and Competitiveness (BES‐2014‐068173) and cofinanced by the European Social Fund. B.S., H.C.B., A.A., C.J., M.J.M., F.V., and N.B. report no disclosures. Y.C. has received funding, research support, and/or honoraria during the past 5 years from UCB, Lundbeck, Medtronic, Abbvie, Novartis, GSK, Boehringer, Pfizer, Merz, Piramal Imaging, and Esteve.

## Supporting information

Additional Supporting Information may be found in the online version of this article at the publisher's web‐site

Supplementary Information
