# Supplementary material for: Patterns of cortical thinning in nondemented Parkinson's disease patients
Source: Mov Disord. 2016 Apr 20;31(5):699–708. doi: 10.1002/mds.26590 (PMC5061099; doi:10.1002/mds.26590)
Supplement: Supplementary file 1 — Supplementary Information [file MDS-31-699-s001.doc]

*Supplementary material*

**Supplementary Methods 1. Neuropsychological assessment**

Visuospatial and visuoperceptual functions were assessed with Benton Visual Form Discrimination (VFD) and Judgment of Line Orientation (JLO) tests; executive functions were evaluated with phonemic (words beginning with the letter “p” in 1 minute) and semantic (animals in 1 minute) fluencies; memory through total learning recall (sum of correct responses from trial I to trial V) and delayed recall (total recall after 20 min) through scores on Rey’s Auditory Verbal Learning Test (RAVLT). Attention and WM were assessed with Digit Span Forward and Backward, the Stroop Color-Word Test, Symbol Digits Modalities Tests (SDMT) and the Trail Making Test (in seconds), part A (TMTA) and part B (TMTB); and language was assessed by the total number of correct responses in the short version of the Boston Naming Test (BNT).

Initially, z scores for each test and for each subject were calculated based on the control group’s means and standard deviations. Expected z scores adjusted for age, sex, and education for each test and each subject were calculated based on a multiple regression analysis performed in the HC group.^1^ As in a previous study,^2^ the presence of MCI was established if the z score for a given test was at least 1.5 lower than the expected score in at least two tests in one domain, or in at least one test per domain in at least two domains.

**Supplementary Methods 2. Cortical thickness procedures**

The procedures carried out by FreeSurfer include removal of nonbrain data, intensity normalization,^3^ tessellation of the gray matter/white matter boundary, automated topology correction,^4,5^ and accurate surface deformation to identify tissue borders).^6-8^ Cortical thickness is then calculated as the distance between the white and gray matter surfaces at each vertex of the reconstructed cortical mantle.^8^ In our study, results for each subject were visually inspected to ensure accuracy of registration, skull stripping, segmentation, and cortical surface reconstruction. Maps were smoothed using a circularly symmetric Gaussian kernel across the surface with a full width at half maximum of 15 mm.

**Supplementary Results 1. PD patterns according to cluster analysis at 2 and 4-cluster level solutions.**

At the 2-cluster level, two patterns of cortical atrophy were identified. PD patients included in *pattern 1* (n=30, 34.09%) showed reduced cortical thickness compared with HC in lateral and medial regions bilaterally, including the precentral gyrus, inferior and superior parietal lobules, cuneus, posterior cingulate gyrus and parahippocampal gyrus. As patients included in *pattern 1* were significantly older and less educated than HC (age mean ± SD for *pattern 1*: 70.60 ± 9.6; for HC: 64.3 ± 8.5, F=11.115, p<0.0001; education mean ± SD for *pattern 1*: 7.77 ± 4.8; for HC: 11.0 ± 4.2, F=8.089; p=0.001), age and years of education were controlled for when comparing these groups. Patients in *pattern 2* (n=58, 65.10%) showed reduced cortical thickness in the left lateral occipital region, and bilaterally in the cuneus and medial orbitofrontal areas in comparison with HC.

Demographical and clinical characteristics of the two patterns are in **supplementary Table 2**, neuropsychological tests results in **supplementary Table 3** and the emotion recognition task in **supplementary Table 4**.

At the 4-cluster level, *pattern 1* was divided into two different clusters. As the number of subjects in one group was too small (n=9), we did not perform additional analyses.

**Supplementary Results 2. Principal Component Analysis**

Eigenvalues extracted from the analyses had the highest separation between the first four components. We chose the first two components because they had the highest separation and explained 12.84% of variance (7.49% the first component and 5.35% the second). The third and fourth components only accounted for 3.74% and 2.95% of the explained variance based on the loadings of the PCA. The first component (x-axis) captured the variability of cortical thickness differences in *pattern 1.* PD patients that had positive loading for the first component were also classified in *pattern 1* (n=25). PD patients who are represented in the PCA plot as negative loading for the first component were from *pattern 2* at the 2 cluster level (n=47). Half of them also had negative loadings for the second component, whereas others were positive. Patients who are represented in the left inferior side of the plot (negative for both components) were those that at the 3 cluster level had no cortical thickness differences with HC (*pattern 3;* n=21). Patients in *pattern 2* at the 3 cluster level had positive loading for the second component (n=26) (**Figure A1**).

**References**

1. Aarsland D, Brønnick K, Larsen JP, Tysnes OB, Alves G. Cognitive impairment in incident, untreated Parkinson disease: the Norwegian ParkWest study. Neurology 2009;72(13):1121-1126.

2. Segura B, Baggio HC, Marti MJ, et al. Cortical thinning associated with mild cognitive impairment in Parkinson’s disease. Mov Disord 2014;29(12):1495-1503.

3. Fischl B, Liu A, Dale AM. Automated manifold surgery: constructing geometrically accurate and topologically correct models of the human cerebral cortex. IEEE Trans Med Imaging 2001;20:70-80.

4. Dale AM, Fischl B, Sereno MI. Cortical surface-based analysis. I. Segmentation and surface reconstruction. Neuroimage 1999;9:179-194.

5. Segonne F, Pacheco J, Fischl B. Geometrically accurate topology-correction of cortical surfaces using nonseparating loops. IEEE Trans Med Imaging 2007;26:518-529.

6. Dale AM, Sereno MI. Improved localization of cortical activity by combining EEG and MEG with MRI cortical surface reconstruction: a linear approach. J Cogn Neurosci 1993;5:162-176.

7. Fischl B, Dale AM. Measuring the thickness of the human cerebral cortex from magnetic resonance images. Proc Natl Acad Sci U S A 2000;97:11050-11055.

8. Fischl B, Salat DH, Busa E, et al. Whole brain segmentation: automated labeling of neuroanatomical structures in the human brain. Neuron 2002;33:341-355.

**Supplementary Table 1** Demographic and clinical characteristics of the sample

|  | **PD (n =88)** | **HC (n = 31)** | **Stats (p value)** |
| --- | --- | --- | --- |
|
| **Sex, male, n (%)** | 51 (58.0) | 16 (51.6) | 0.375 (0.540)^a^ |
| **Age, y** | 64.1 ± 10.6 | 64.3 ± 8.5 | 0.099 (0.921)^b^ |
| **Education, y** | 10.6 ± 5.3 | 11.0 ± 4.2 | 0.417(0.677)^b^ |
| **MMSE** | 29 ± 1.1 | 29.7 ± 0.5 | 4.36 (<0.0001)^b^ |
| **Disease duration, y** | 8 ± 5.7 | NA | NA |
| **Age of onset, y** | 56.12 ± 11.6 | NA | NA |
| **Early PD, 5 y, (%)** | 37 (42.0) | NA | NA |
| **BDI** | 10.77 ± 6.3 | 6.03 ± 5.7 | -3.582 (0.001)^b^ |
| **Apathy** | 12.70 ± 7.2 | 8.38 ± 5.1 | -3.497 (0.001)^b^ |
| **NPI** | 5.74 ± 7.5 | 1.52 ± 3.2 | -4.183 (<0.0001)^b^ |
| **Visual Hallucinations, n (%)** | 17 (19.8) | 0 (0.0) | NA |
| **UPDRS part III** | 15.5 ± 9.9 | NA | NA |
| **Hoehn & Yahr stage, n**  **1/1.5/2/2.5/3** | 22/5/43/8/10 | NA | NA |
| **LEDD, mg** | 803.93 ± 494 | NA | NA |

Abbreviations: BDI = Beck Depression Inventory; HC = Healthy Controls; LEDD = L-Dopa Equivalent Daily Dose; MMSE = Mini-Mental State Examination; NA = not applicable; NPI = Cumming’s Neuropsychiatric Inventory; PD = Parkinson’s disease; UPDRS III = Unified Parkinson’s Disease Rating Scale III.

Data are presented as mean ± SD.

1. The Chi-test was used.
2. T-student test was used.

Supplementary Table 2 Demographic and clinical characteristics at 2-cluster level

|  | **PD subtypes** | | | **HC (n = 31)** | | **Stats (p value)** |
| --- | --- | --- | --- | --- | --- | --- |
|  | **Pattern 1 (n=30)** | | **Pattern 2 (n=58)** |  |  |  |
| **Sex, male, n (%)** | 15 (50.0) | | 36 (62.1) | 16 (51.6) | | 1.545 (0.462)^a^ |
| **Age, y** | 70.60 ± 9.6 | | 60.76 ± 9.5 | 64.3 ± 8.5 | | 11.115 (<0.0001)^c,e,g^ |
| **Education, y** | 7.77 ± 4.8 | | 12.05 ± 5.0 | 11.0 ± 4.2 | | 8.089 (0.001)^c,e,g^ |
| **MMSE** | 28.57 ± 1.4 | | 29.28 ± 0.9 | 29.7 ± 0.5 | | 10.462 (<0.0001)^d,e,f,g^ |
| **Disease duration, y** | 8.77 ± 6.6 | | 7.60 ± 5.1 | NA | | 0.917 (0.362)^b^ |
| **Age of onset, y** | 61.83 ± 12.7 | | 53.16 ± 9.9 | NA | | 3.252 (0.002)^b^ |
| **Early PD, <=5 y, (%)** | 12 (40.0) | | 25 (43.1) | NA | | 0.078 (0.780)^a^ |
| **BDI** | 13.67 ± 5.7 | | 9.27 ± 6.2 | 6.03 ± 5.7 | | 11.824 (<0.0001)^c,e,g^ |
| **Apathy** | 15.11 ± 7.9 | | 11.43 ± 6.5 | 8.38 ± 5.1 | | 7.489 (0.001)^d,e^ |
| **NPI** | 6.59 ± 7.8 | | 5.31 ± 7.4 | 1.52 ± 3.2 | | 4.352 (0.015)^d,f^ |
| **Visual Hallucinations, n (%)** | 6 (20.0) | | 11 (19.6) | 0 (0.0) | | 7.640 (0.106)^a^ |
| **UPDRS part III** | 18.07 ± 9.1 | | 14.12 ± 10.1 | NA | | 1.798 (0.076)^b^ |
| **Hoehn & Yahr stage, n**  **1/1.5/2/2.5/3** | 2/3/16/4/5 | | 20/2/27/4/5 | NA | | 9.827 (0.043)^a^ |
| **LEDD,mg** | 764.63 ± 388.28 | | 824.26 ± 542.72 | NA | | -0.593 (0.555)^b^ |
| **Total MCI, n (%)** | | 20 (66.7) | 25 (43.1) | NA | 4.394 (0.036)^a^ | |
| **Visuospatial functions, n (%)** | | 10 (33.3) | 16 (27.6) | NA | 0.314 (0.575)^a^ | |
| **Executive functions, n (%)** | | 16 (3.3) | 12 (20.7) | NA | 9.712 (0.002)^a^ | |
| **Memory, n (%)** | | 14 (46.7) | 20 (34.5) | NA | 1.238 (0.266)^a^ | |
| **Attention and WM, n (%)** | | 20 (66.7) | 31 (53.4) | NA | 1.418 (0.234)^a^ | |
| **Language, n (%)** | | 2 (6.7) | 5 (8.6) | NA | 0.103 (0.748)^a^ | |

Abbreviations: Apathy = Starkstein’s Apathy Scale; BDI = Beck Depression Inventory-II; HC = Healthy Controls; LEDD = L-DOPA Equivalent Daily Dose; MCI = Mild Cognitive Impairment; MMSE = Mini-Mental State Examination; NA = not applicable; NPI = Cumming’s Neuropsychiatric Inventory; PD = Parkinson’s disease; UPDRS III = Unified Parkinson’s Disease Rating Scale, motor section; WM = Working Memory.

Data are presented as mean ± SD(continuous) or frequencies (categorical).

1. The Chi-test was used.
2. T-student test was used.
3. Analysis of variance (ANOVA) followed by Bonferroni post hoc test was used.
4. Analysis of variance (ANOVA) followed by Tamhane (T2) post hoc test was used.
5. Significant differences (p<0.05) between HC and Pattern 1.
6. Significant differences (p<0.05) between HC and Pattern 2.
7. Significant differences (p<0.05) between both PD patterns.

Supplementary Table 3 Neuropsychological tests results at the 2 cluster level solution

|  | **PD subtypes** | |  |  |
| --- | --- | --- | --- | --- |
|  | **Pattern 1 (n=30)** | **Pattern 2 (n=58)** | **HC (n=31)** | **Stats (p value)** |
| ***Visuospatial functions*** | | | | |
| **VFD** | -0.83 ± 1.3 | -0.59 ± 1.1 | -0.04 ± 0.9 | 4.012 (0.021)^a,c^ |
| **JLO** | -0.67 ± 0.9 | -0.45 ± 1.1 | 0.06 ± 0.7 | 4.659 (0.011)^a,c^ |
| ***Executive functions*** | | | | |
| **Phonetic fluency** | -0.46 ± 1.0 | -0.11 ± 1.1 | 0.01 ± 0.9 | 1.834 (0.164)^a^ |
| **Semantic fluency** | -1.37 ± 1.0 | -0.18 ± 1.3 | 0.07 ± 1.0 | 14.095 (<0.0001)^a,c,e^ |
| ***Memory*** | | | | |
| **RAVLT total** | -1.19 ± 1.5 | -0.46 ± 1.3 | 0.05 ± 0.9 | 7.437 (0.001)^b,c^ |
| **RAVLT recall** | -0.88 ± 1.1 | -0.54 ± 1.3 | 0.02 ± 0.9 | 4.415 (0.014)^b,c^ |
| ***Attention and WM*** | | | | |
| **Span digits forward** | 0.11 ± 0.9 | -0.26 ± 0.9 | 0.03 ± 0.8 | 2.084 (0.129)^a^ |
| **Span digits backward** | 0.11 ± 0.7 | -0.08 ± 0.9 | 0.02 ± 0.9 | 0.467 (0.628)^a^ |
| **Stroop Word Test** | -0.89 ± 1.2 | -0.93 ± 1.3 | 0.01 ± 0.9 | 6.762 (0.002)^a,c,d^ |
| **Stroop Color Test** | -0.49 ± 0.9 | -0.21 ± 0.8 | 0.08 ± 0.9 | 3.599 (0.031)^a,c^ |
| **Stroop Word-Color Test** | -0.34 ± 0.7 | -0.08 ± 0.8 | 0.13 ± 0.7 | 2.783 (0.066)^a^ |
| **SDMT** | -1.05 ± 0.9 | -0.59 ± 0.9 | 0.01 ± 0.6 | 11.078 (<0.0001)^a,c,d^ |
| **TMT-A** | 2.09 ± 3.3 | 0.97 ± 3.0 | -0.06 ± 0.8 | 4.827 (0.010)^b,c^ |
| **TMT-B** | 1.72 ± 2.8 | 0.98 ±1.9 | -0.09 ± 0.6 | 6.296 (0.003)^b,c,d^ |
| **TMT A minus B** | -1.80 ± 3.2 | -0.95 ± 1.9 | 0.14 ± 0.7 | 6.450 (0.002)^b,c,d^ |
| ***Language*** | | | | |
| **BNT** | -0.07 ± 1.2 | -0.24 ± 0.9 | 0.16 ± 0.7 | 1.776 (0.174)^a^ |

Abbreviations: BNT = Boston Naming Test; HC = Healthy Controls; JLO = Judgement of Line Orientation; PD = Parkinson’s disease; RAVLT = Rey’s Auditory and Verbal Learning Test; SDMT = Symbol Digits Modalities Test; TMT = Trail Making Test; VFD = Visual Form Discrimination; WM = working memory.

Data are presented as mean ± SD. All scores are z scores.

1. Analysis of variance (ANOVA) followed by Bonferroni post hoc test was used.
2. Analysis of variance (ANOVA) followed by Tamhane (T2) post hoc test was used.
3. Significant differences (p<0.05) between HC and Pattern 1.
4. Significant differences (p<0.05) between HC and Pattern 2.
5. Significant differences (p<0.05) between both PD patterns.

Supplementary Table 4 Results from emotion recognition tests at the 2-cluster level

|  | **PD subtypes** | | **HC (n=31)** | **Test stats (p value)** |
| --- | --- | --- | --- | --- |
|  | **Pattern 1 (n=30)** | **Pattern 2 (n=58)** |  |  |
| **Ekman anger** | -0.23 ± 1.0 | -0.11 ± 0.9 | 0.07 ± 1.0 | 0.735 (0.482)^a^ |
| **Ekman disgust** | -0.45 ± 1.6 | -0.47 ± 1.0 | 0.09 ± 0.9 | 2.266 (0.109)^b^ |
| **Ekman fear** | 0.00 ± 0.8 | 0.01 ± 0.9 | -0.07 ± 1.0 | 0.085 (0.918)^a^ |
| **Ekman sadness** | -0.19 ± 1.1 | -0.39 ± 0.8 | 0.14 ± 0.7 | 3.345 (0.039)^a,c^ |
| **Ekman happy** | -0.18 ± 1.6 | -0.39 ± 1.5 | -0.11 ± 1.0 | 0.389 (0.679)^a^ |
| **Ekman surprise** | -0.40 ± 1.4 | -0.02 ± 1.0 | 0.04 ± 0.9 | 1.408 (0.249)^a^ |
| **Ekman total score** | -0.12 ± 0.7 | -0.00 ± 0.5 | 0.02 ± 1.1 | 0.285 (0.753)^a^ |

Results of the Ekman 60 Faces Test, presented in z scores.

Abbreviations: HC = healthy controls; PD = Parkinson’s Disease.

1. Analysis of variance (ANOVA) followed by Bonferroni post hoc test.
2. Analysis of variance (ANOVA) followed by Tamhane (T2) post hoc test.
3. Significant differences (p<0.05) between HC and Pattern 2.

Supplementary Table 5 Neuropsychological tests results at the 3 cluster level solution

|  | **PD subtypes** | | |  |  |
| --- | --- | --- | --- | --- | --- |
|  | **Pattern 1 (n=30)** | **Pattern 2 (n=29)** | **Pattern 3 (n=29)** | **HC (n=31)** | **Stats (p value)** |
| ***Visuospatial functions*** | | | | | |
| **VFD** | -0.83 ± 1.3 | -0.55 ± 1.0 | -0.62 ± 1.2 | -0.04 ± 0.9 | 2.674 (0.051)^a,c^ |
| **JLO** | -0.67 ± 0.9 | -0.65 ± 1.2 | -0.26 ± 1.0 | 0.06 ± 0.7 | 3.933 (0.010)^a,c,d^ |
| ***Executive functions*** | | | | | |
| **Phonetic fluency** | -0.46 ± 1.0 | -0.05 ± 1.1 | -0.17 ± 1.1 | 0.01 ± 0.9 | 1.280 (0.285) |
| **Semantic fluency** | -1.37 ± 1.0 | -0.16 ± 1.1 | -0.21 ± 1.5 | 0.07 ± 1.0 | 9.325 (<0.0001)^a,c,f,g^ |
| ***Memory*** | | | | | |
| **RAVLT total** | -1.19 ± 1.5 | -0.55 ± 1.3 | -0.37 ± 1.3 | 0.05 ± 0.9 | 5.031 (0.003)^a,c^ |
| **RAVLT recall** | -0.88 ± 1.1 | -0.64 ± 1.4 | -0.44 ± 1.3 | 0.02 ± 0.9 | 3.061 (0.031)^a,c^ |
| ***Attention and WM*** | | | | | |
| **Span digits forward** | 0.11 ± 0.9 | -0.55 ± 0.8 | 0.04 ± 0.9 | 0.03 ± 0.8 | 3.687 (0.014)^a,f^ |
| **Span digits backward** | 0.11 ± 0.7 | -0.25 ± 0.8 | 0.10 ± 1.0 | 0.02 ± 0.9 | 1.124 (0.342) |
| **Stroop Word Test** | -0.89 ± 1.2 | -0.99 ± 1.4 | -0.88 ± 1.4 | 0.01 ± 0.9 | 4.506 (0.005)^a,c,d,e^ |
| **Stroop Color Test** | -0.49 ± 0.9 | -0.38 ± 0.8 | -0.05 ± 0.7 | 0.08 ± 0.9 | 3.149 (0.028)^a,c^ |
| **Stroop Word-Color Test** | -0.34 ± 0.7 | -0.18 ± 0.9 | 0.02 ± 0.7 | 0.13 ± 0.7 | 2.161 (0.097) |
| **SDMT** | -1.05 ± 0.9 | -0.70 ± 1.0 | -0.48 ± 0.8 | 0.01 ± 0.6 | 7.685 (0.000)^a,c,d^ |
| **TMT-A** | 2.09 ± 3.3 | 1.44 ± 3.8 | 0.49 ± 1.7 | -0.06 ± 0.8 | 3.848 (0.011)^b,c^ |
| **TMT-B** | 1.72 ± 2.8 | 1.04 ± 1.6 | 0.93 ± 2.2 | -0.09 ± 0.6 | 4.178 (0.008)^b,c,d^ |
| **TMT A minus B** | -1.80 ± 3.2 | -0.98 ± 1.6 | -0.91 ± 2.1 | 0.14 ± 0.7 | 4.267 (0.007)^b,c,d^ |
| ***Language*** | | | | | |
| **BNT** | -0.07 ± 1.2 | -0.35 ± 0.9 | -0.13 ± 0.9 | 0.16 ± 0.7 | 1.448 (0.233) |

Abbreviations: BNT = Boston Naming Test; HC = Healthy Controls; JLO = Judgement of Line Orientation; PD = Parkinson’s disease; RAVLT = Rey’s Auditory and Verbal Learning Test; SDMT = Symbol Digits Modalities Test; TMT = Trail Making Test; VFD = Visual Form Discrimination; WM = working memory.

Data are presented as mean ± SD. All scores are z scores.

1. Analysis of variance (ANOVA) followed by Bonferroni post hoc test.
2. Analysis of variance (ANOVA) followed by Tamhane (T2) post hoc test.
3. Significant differences (p<0.05) between HC and Pattern 1.
4. Significant differences (p<0.05) between HC and Pattern 2.
5. Significant differences (p<0.05) between HC and Pattern 3.
6. Significant differences (p<0.05) between Pattern 1 and Pattern 2.
7. Significant differences (p<0.05) between Pattern 1 and Pattern 3.

**
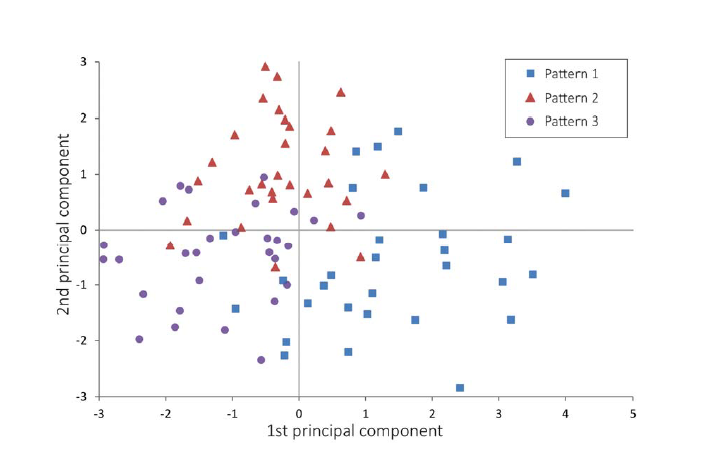
**

**Supplementary Figure 1 Principal component analysis results and distribution according to the 3 PD patterns**.

*Graphics program:* Microsoft Excel® and edited with Adobe Photoshop®.
